# Clinical Teaching—the New Amphitheater at the Hospital

**Published:** 1849-09

**Authors:** 


CLINICAL TEACHING—THE NEW AMPHITHEATER AT THE HOSPITAL.

We congratulate the friends of medical science, throughout the West and South, on the auspicious prospects of clinical teaching, in the city of Louisville. The City Council have ordered the erection of a building, in connection with the hospital, which will be all that can be desired for the purpose. The contractors are now at work, and are pushing the building forward, so energetically that there is no doubt of its completion, by the time provided in the contract—the 20th of October.

This new Amphitheater is at the eastern end of the Louisville Marine Hospital, and communicates with the wards of that institution. Its form is octagonal. The diameter of the lecture room is fifty feet, and the height of the ceiling is twenty feet. The space for the rostrum is ten by twenty-two feet. The room is abundantly lighted by an ample sky-light. The seats, instead of ascending in a straight line from the rostrum, ascend in a concave, somewhat resembling the concavity of a bowl. The advantages of this arrangement are obvious—in such an arrangement there can be but little choice of seats, for the highest, or the one farthest from the rostrum, affords as much facility for seeing all that is essential to be seen, as the lowest one does. There is but one other amphitheater of this kind, that we know of, and that is in the Royal Institution in London. That one has given so much satisfaction to auditors, spectators and teachers, that it is strange, that its advantages have not been more frequently imitated.

The Amphitheater of the Marine Hospital will very conveniently seat four hundred students, and offers abundant attractions to all who desire to enjoy, what must be the basis of all successful medical teaching—Clinical instruction. In this department of medical education, the medical school of Louisville will offer to students, in the ensuing session, facilities second to no other in the Union. The celebrity of the Professor of the Theory and Practice of Medicine—Professor Bartlett—is so widely spread, that it is unnecessary for us to dilate upon the services he will be able to render his classes, in the fields of clinical instruction. In this department he certainly has no superior in this country, and his connection with the school of Louisville, possessing as it does, the most ample means for clinical teaching, is destined to exert a very happy influence upon the interests of western medicine.

We are gratified to hear, that under the present arrangements of the hospital, the internal affairs of the institution are in a highly prosperous condition. We are assured, from various quarters, that the hospital has never been in as good a condition as it is at present, and there is abundant reason for supposing that these good fruits will rather grow than lessen.

To students at a distance, we beg leave to express our belief that the facilities of the Louisville school for medical instruction have at no time been equal to those now possessed by the institution, and that the session of 1849 and '50 will long be remembered as the commencement of a race of the most honorable usefulness. B.

